# MicroRNA-143 suppresses oral squamous cell carcinoma cell growth, invasion and glucose metabolism through targeting hexokinase 2

**DOI:** 10.1042/BSR20160404

**Published:** 2017-06-07

**Authors:** Xianghui Sun, Lei Zhang

**Affiliations:** 1 Tianjin Key Laboratory of Artificial Cell, Artificial Cell Engineering Technology Research Center of Public Health Ministry, Department of Stomatology, Tianjin Third Central Hospital, Tianjin 300170, P.R. China; 2 Tianjin Key Laboratory of Artificial Cell, Artificial Cell Engineering Technology Research Center of Public Health Ministry, Department of Clinical Laboratory, Tianjin Third Central Hospital, Tianjin 300170, P.R. China

**Keywords:** hexokinase 2, microRNA, miR-143, oral cancer

## Abstract

miRNAs are non-coding RNAs that have functions to regulate gene expression and play essential roles in a variety of biological processes of cancers. In the present study, we report *miR-143* acts as a tumor suppressor in human oral squamous cell carcinoma (OSCC). The expressions of *miR-143* are down-regulated in both OSCC cell lines and patient samples compared with normal adjacent tissues. We found overexpression of *miR-143* in oral cancer cell lines suppresses cell migration, cellular glucose metabolism and proliferation. Moreover, overexpression of *miR-143* promoted apoptosis and significantly caused cell cycle arrest at G_1_ stage. The colony formation of oral cancer cells was also suppressed by *miR-143*. We identified hexokinase 2 (HK2) as a direct target of *miR-143* in oral cancer cells. Our data show that *miR-143* complementary pairs to the 3′-UTR of HK2 in oral cancer cells, leading to the inhibition of glycolysis *in vitro* and *in vivo*. Moreover, knockdown of HK2 by siRNA in oral cancer cells inhibited glucose metabolism, proliferation and migration. Recovery of glucose metabolism by overexpression of HK2 in *miR-143* overexpressing cells restores the cell migration and proliferation, suggesting that the *miR-143*-mediated cancer suppression is through the direct inhibition of HK2. In summary, the present studies highlight *miR-143* as a tumor suppressor in OSCC by the suppression of cell migration, glucose metabolism and proliferation through directly targeting HK2, rendering *miR-143* a therapeutic strategy for the treatment of clinical OSCC patients.

## Introduction

Oral squamous cell carcinoma (OSCC) is the most common head and neck neoplasm, representing 95% of all forms of head and neck cancer and patients with OSCC display a disappointing 5-year survival rate [1,2]. Recently, the clinical outcome of OSCC has gradually improved, however, patients with OSCC develop chemoresistance reflecting limits in our understanding of the pathogenesis of this cancer [3,4]. Therefore, a better understanding of the molecular mechanisms for oral cancer carcinogenesis contributes to development of novel diagnostic and therapeutic approaches to improve the treatments and prognosis of OSCC patients.

miRNAs are a group of short non-coding RNAs (18–22 nt), which negatively regulate protein translation by binding to complementary sequences in the 3′-UTR of target mRNAs [5]. Cumulative studies reported that miRNAs regulate a wide range of biologic processes of cancers such as proliferation, apoptosis, metastasis, chemosensitivity, tumor development, and stem cell maintenance [6,7]. Moreover, abnormalities of miRNAs have been implicated in the pathogenesis of multiple cancers, suggesting either oncogenic or tumor suppressive roles of miRNAs [8]. Therefore, identification of aberrantly expressed miRNAs is an important step toward the study of the functions of miRNAs in oral cancer carcinogenesis.

In particular, *miR-143* has been extensively studied as a tumor suppressor in several cancers such as breast cancer [9], colon cancer [10], lung cancer [11], pancreatic cancer [12], brain tumor [13], and melanoma [14]. However, the roles of *miR-143* and its target gene in regulating human OSCC development are poorly understood. In the present study, the roles of *miR-143* in the human OSCC will be investigated. The potential target of *miR-143* and the regulator mechanisms of *miR-143* in oral cancer proliferation, invasion, and metabolism will be assessed. Our study will contribute to the development of the miRNAs-based therapeutic agents for the clinical treatments of oral cancer patients.

## Materials and methods

### Cell culture and tissue specimens

The collection of tumor specimens from OSCC patients was approved by the Institutional Review Board (IRB) of Tianjin Third Central Hospital. Human oral cancer cell lines (OECM-1 and Tca8113) were obtained from the cell bank of type culture collection of Chinese Academy of Sciences (Shanghai, China). Cells were routinely cultured in Dulbecco’s modified Eagle’s medium (DMEM; Gibco BRL, Paisley, U.K.) containing 10% FBS (HyClone, Logan, UT, U.S.A.), and 100 units/ml penicillin, 100 mg/ml streptomycin (HyClone, Logan, UT, U.S.A.) at 37°C in a humid atmosphere with 5% CO_2_.

### miRNAs and plasmid DNA transfection

*miR-143* mimic and control mimic were obtained from ThermoFisher Scientific (Waltham, MA, U.S.A.). The miRNAs and plasmid DNA for overexpressing HK2 (hexokinase 2) were transfected using Lipofectamine® 2000 (Invitrogen Life Technologies). After 48 h following transfection, the expression of *miR-143* was detected by quantitative-reverse transcription polymerase chain reaction (qRT-PCR), and the expression of hexokinase was measured by Western blotting.

### Luciferase assays

The 3′-UTR luciferase vector was constructed using the pMIR-report luciferase vector containing wild-type or mutant 3′-UTR of *HK2* mRNA, which carries a putative *miR-143* complementary site. OECM-1 and Tca8113 cells (3 × 10^4^ per well) were pre-seeded in a 24-well plate the day before transfection for overnight. Cells were transfected with 0.5 μg of the 3′-UTR luciferase vector and 50 nM *miR-143* mimics or negative control using Lipofectamine RNAiMAX (Invitrogen). Assays were performed using the pMIR-report luciferase vector system after 48 h of co-transfection.

### Cell proliferation assay

The cancer cells were transfected with *miR-143* mimic, or control mimic for 48 h. Cells were then seeded in a 96-well plate, at a density of 3000 cells/well for overnight incubation. The cell proliferation rates were measured with MTT assay (Sigma–Aldrich, Inc., St. Louis, MO, U.S.A.). Briefly, cells were treated with MTT at 50 mg per well. The generated formazan was dissolved in DMSO, and the absorbance was recorded by measuring the absorbance at 590 nm with a plate reader. The same experiment was repeated three times.

### Scratch and cell migration assays

For wound-healing assays, 1 × 10^5^ cells were seeded on glass coverslips and cultured until confluence. Cells were scratched with micropipette tips, and images were captured at 0 and 24 h after wounding. The transwell assay was done by using a transwell chamber consisting of 8 mm membrane filter inserts (Corning, Corning, NY, U.S.A.) according to the previous description [15]. For each experiment, the number of cells in three random fields on the underside of the filter was counted, and three independent filters were analyzed.

### Cell cycle analysis

The cell cycle was analyzed using the Cell Cycle Assay Kit (Abcam, #ab112116) according to the manufacturer’s protocol.

### Colony formation assay

One hundred oral cancer cells were placed into each 6 cm cell culture dish and cultured for 2 weeks for allowing the colony formation. Cells were fixed by methanol and stained by 0.05% Crystal Violet for 5 min. After washing by PBS, the dishes were recoded.

### Measurements of glucose metabolism

Glucose uptake was detected using a Glucose Uptake Colorimetric Assay Kit (#K676-100, BioVision, Inc., Milpitas, CA, U.S.A.) and lactate production was detected using a Lactate Colorimetric Assay Kit II (#K627-100, BioVision, Inc., Milpitas, CA, U.S.A.) according to the manufacturer’s instructions. Results were normalized by the protein quantities of experimental group to the control group. All experiments were repeated three times.

### LDH activity assay

The LDH activity assay was performed using the Lactate Dehydrogenase Activity Assay Kit (Sigma, # MAK066, St. Louis, MO, U.S.A.) according to the manufacturer’s protocol.

### Xenograft experiments

Athymic nude mice were purchased from Vital River (Beijing, China) and housed in the certified animal facility in the Tianjin Third Central Hospital. All experimental procedures were performed according to the Guide for the Care and Use of Laboratory Animals published by the U.S. National Institutes of Health (NIH Publication No. 85-23, revised 1996). For developing human oral cancer xenografts, OECM-1 cells (3 × 10^6^) in were implanted by subcutaneous (s.c.) injection into each mouse. After tumor development, the mice were randomly divided into two groups: control miRNA and *miR-143* mimic injection. The injection was given twice a week for 2 weeks. Tumors were taken after injections for the downstream experiments.

### Tissue immunohistochemistry

Human oral tumor IHC was performed according to previous descriptions [16]. Briefly, sections of *miR-143* low or high expression were deparaffinized in xylene. Then, samples were rehydrated through a gradient concentration of alcohol followed by incubation by 5% normal goat serum to block non-specific staining. Sections were then incubated with rabbit anti-HK2 antibody (1:200, Cellsignaling, #2867, Danvers, MA, U.S.A.) overnight at 4°C. The slides were incubated with biotin-labeled goat anti-rabbit IgG and further incubated with streptavidin peroxidase solution (SABC kit, Boster Biological Technology, Ltd., Wuhan, China). The staining was visualized by reaction with 3,3′-di-aminobenzidine (Boster Biological Technology, Ltd.) in PBS for 5 min at room temperature. All of the IHC staining results were reviewed independently by two pathologists.

### qRT-PCR

Total RNA was extracted from cells or human OSCC tumor tissues using TRIzol reagent (Invitrogen) according to the manufacturer’s protocol. Total RNA was used for reverse transcription using the PrimerScript RT-PCR kit (TaKaRa Biotechnology, Dalian, China). miRNAs were reverse transcribed using sequence-specific stem-loop primers (Invitrogen). Quantitative RT-PCR was conducted according to the previous description [15]. Briefly, qRT-PCR was conducted using a standard SYBR Green PCR kit (Roche) protocol with a CFX96 Touch™ Real-Time PCR Detection System (Biorad). The relative expression was calculated using the 2−ΔΔ^*C*_t_^ method. The transcription levels of GAPDH or U6 were used as an internal control. The specific Primers are listed as follows: GAPDH: forward: 5′-GCACCGTCAAGGCTGAGAAC-3′; reverse: 5′-TGGTGAAGACGCCAGTGGA-3′; HK2: forward: 5′-CAAAGTGACAGTGGGTGTGG-3′; reverse: 5′-GCCAGGTCCTTCACTGTCTC-3′.

### Western blotting

Cells were collected and lysed using RIPA buffer (Pierce, Waltham, MA, U.S.A.) followed by protein concentration measurements by Bradford assay. Equal amount of protein samples were separated by 10% SDS/PAGE, and were electrophoretically transferred on to a PVDF membrane (Millipore Corporation). The membrane was blocked with 5% low-fat milk in Tris buffered saline for 1 h at room temperature, and then incubated with primary antibodies (β-actin and HK2: cell signaling) at 1:1000 dilution at 4°C overnight. The primary antibodies were detected with horseradish peroxidase-conjugated secondary antibodies and a chemiluminescence detection kit (Pierce, Waltham, MA, U.S.A.).

### Statistical analysis

The statistical significance was assessed by Student’s *t* test using GraphPad Prism program 5.0 (GraphPad Software, U.S.A.). *P*<0.05 was considered statistically significant.

## Results

### *miR-143* levels are significantly down-regulated in human OSCC tumors

**Figure 1:**
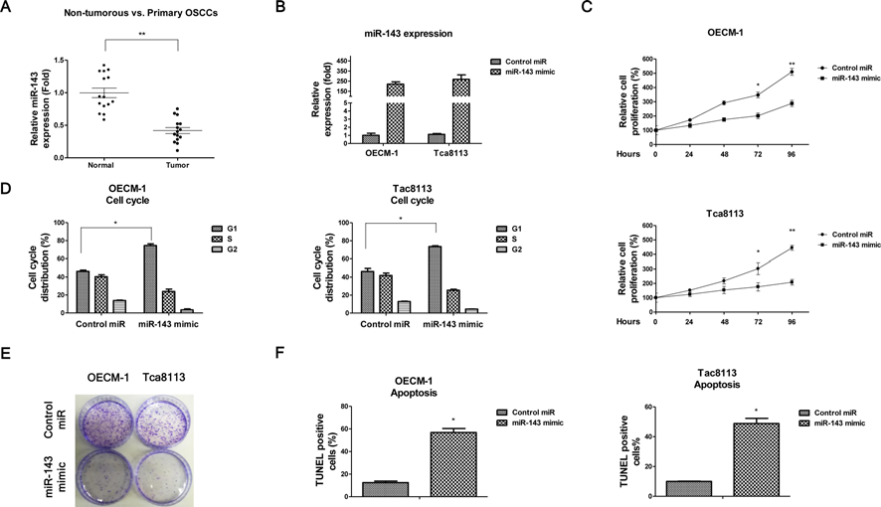
*miR-143* is downregulated in patient OSCC species and suppress oral cancer cells growth (**A**) Expression level of *miR-143* in each individual case of OSCCs and adjacent normal tissues was measured by qRT-PCR. snRNA U6 was used as an internal control. (**B**) OECM-1 and Tca8113 cells were transfected with *miR-143* mimic or control miRNAs for 48 h, the expressions of *miR-143* were measured by qRT-PCR. (**C**) OECM-1 and Tca8113 cells were transfected with miR-143 mimic or control miRNAs for 48 h, cells were plated into 24-well plate and the cell proliferation rates were measured at 0, 24, 48, 72, and 96 h by MTT assay. (**D**) The transfected OECM-1 and Tac8113 cells were subjected to cell cycle analysis, (**E**) colony formation assay and (**F**) apoptosis assay. Data are presented as mean ± S.D.: *, *P*<0.05; **, *P*<0.01.

Previous studies revealed that *miR-143* possesses tumor-suppression function in multiple tumor types [9–14]. To evaluate the function of *miR-143* in human oral tumor tissues, we analyzed the expressions of *miR-143* from 15 human normal health oral tissues and 15 human oral cancer tissues using real-time PCR. As we expected, the expression levels of *miR-143* in OSCC tumor species were significantly lower than that in normal oral tissues (Figure 1A), suggesting *miR-143* act as a tumor suppressor in OSCC.

### *miR-143* suppresses OSCC cells proliferation, cell cycle and colony formation, promotes apoptosis

To further validate the tumor-suppression functions of miR-143 in OSCC, the effects of miR-143 on cell invasion, glucose metabolism, and tumor proliferation were measured. We transiently transfected miR-143 mimic into two human OSCC cell lines, OECM-1 and Tca8113 (Figure 1B). Overexpression of miR-143 markedly suppressed the proliferation ratios of these two oral cancer cell lines (Figure 1C) compared with negative miRNA transfection. Moreover, we found overexpression of *miR-143* caused a cell cycle arrest of oral cancer cells in G_1_-phase (Figure 1D). The colony formation assay demonstrated that overexpression of *miR-143* significantly suppressed colony of OCEM-1 and Tca8113 (Figure 1E). The apoptosis was induced by *miR-143* (Figure 1F).

### *miR-143* suppresses OSCC cells migration, invasion and glucose metabolism

**Figure 2:**
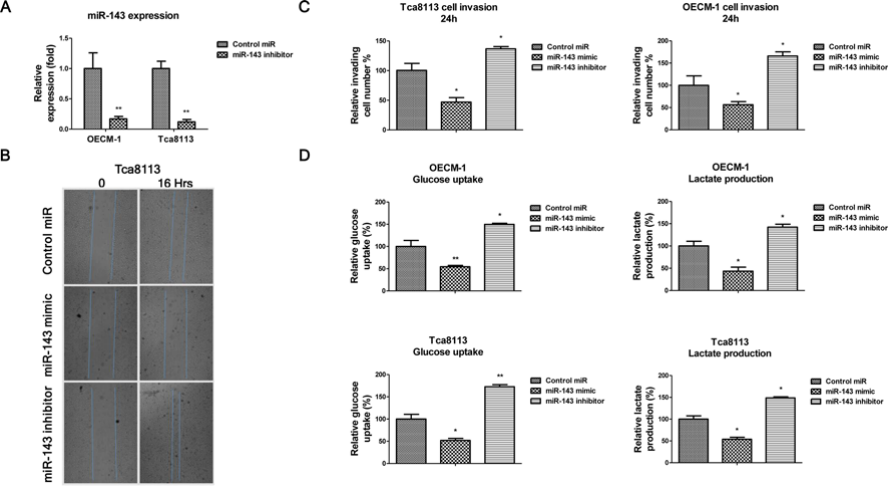
*miR-143* inhibits *in vitro* migration, invasion, and glucose metabolism of oral cancer cells (**A**) OECM-1 and Tac8113 cells were transfected with negative control or miR-143 inhibitor for 48 h, the expression of *miR-143* was measured by qRT-PCR. (**B**) Wound healing assay showed the migration ability of Tca8113 cells with *miR-143* mimic transfection was significantly lower than the Tca8113 cells with control mimic transfection. The migration ability of Tca8113 cells with *miR-143* inhibitor transfection was significantly higher than the Tca8113 cells with control mimic transfection at 16 h. (**C**) Transwell assay showed the cell invasion abilities of Tca8113 (left) and OECM-1 were significantly inhibited by *miR-143* mimic transfection. (**D**) OECM-1 (upper) and Tca8113 (lower) cells were transfected with control mimic, *miR-143* mimic or *miR-143* inhibitor for 48 h. The glucose uptake (left) and lactate production (right) were compared. Data are presented as mean ± S.D. *, *P*<0.05; **, *P*<0.01.

In addition, the invasive abilities of oral cancer cells were significantly inhibited by *miR-143* but promoted by *miR-143* inhibitor (Figure 2A–C). It has been reported that dysregulated glycolysis reflected one of the features of cancer cells [17], we measured the glucose uptake and lactate production both of which are indicators of glucose metabolism. Consistently, the glucose metabolism was attenuated by overexpression of *miR-143* but activated by *miR-143* inhibition (Figure 2D), supporting the above results that *miR-143* may play a suppressive role in oral cancer.

### HK2 is a direct target of *miR-143* in OSCC cells

**Figure 3:**
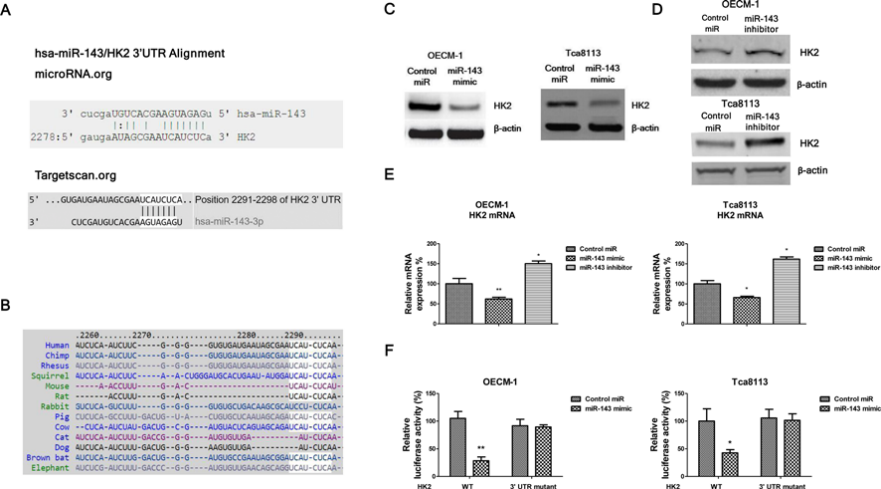
*miR-143* directly targets HK2 in oral cancer cells (**A**) The predicted complimentary binding of *miR-143* on the 3′-UTR of HK2 by microRNA.org (top) and Targetscan.org (bottom). (**B**) Conserved binding sequence of *miR-143* were found in multiple species. (**C**) OECM-1 (left) and Tca8113 (right) cells were transfected with control mimic or *miR-143* mimic for 48 h, then cells were collected and subjected to Western blot analysis. β-Actin was a loading control. (**D**) OECM-1 (top) and Tca8113 (bottom) cells were transfected with negative control or *miR-143* inhibitor for 48 h, then cells were collected and subjected to Western blot analysis. β-Actin was a loading control. (**E**) OECM-1 (left) and Tca8113 (right) cells were transfected with control, *miR-143* mimic or *miR-143* inhibitor, then the HK2 mRNAs were measured by qRT-PCR. (**F**) OECM-1 (left) and Tca8113 (right) cells were co-transfected with control, *miR-143* mimic and luciferase reporter vector containing wild type or mutant 3′-UTR of HK2 for 48 h, followed by the luciferase assay analysis. Data are presented as mean ± S.D.: *, *P*<0.05; **, *P*<0.01.

To investigate the mechanisms by which *miR-143* suppresses OSCC cells proliferation, invasion and glucose metabolism, we searched the potential targets of *miR-143* by the web-based target analysis tools www.microRNA.org and www.targetscan.org. The analysis software predicted that HK2 might be a target for *miR-143* and the 3′-UTR of HK2 contains a highly conserved binding site for *miR-143* (Figure 3A). In addition, the binding of *miR-143* on HK2 3′-UTR is conserved in multiple species (Figure 3B), further supporting our results that *miR-143* directly targets HK2 and this binding has important functions. Currently, there is no publication reported that HK2 was a *miR-143* direct target in OSCC cells. To determine whether *miR-143* could target HK2 in endothelial cells, we transfected control miRNA, *miR-143* mimic or *miR-143* inhibitor into OECM-1 and Tca8113 cells. Our results showed overexpression of *miR-143* inhibited HK2 protein and mRNA levels and inhibition of *miR-143* could increase HK2 protein and mRNA levels (Figure 3C–E). To further validate targeting of HK2 by *miR-143*, we investigated whether *miR-143* directly interacted with the 3′-UTR of HK2 using a dual-luciferase reporter assay. A reporter plasmid harboring a mutated *miR-143* binding site was used as a control. Overexpression of *miR-143* significantly suppressed luciferase activity of 3′-UTR of the wild-type HK2 reporter constructs in both OECM-1 and Tca8113 cells (Figure 3F), while the suppressive effect of *miR-143* mimic was abrogated with mutant HK2 3′-UTR. These results demonstrated that HK2 was indeed a direct downstream target of *miR-143* in human OSCC cells.

### Knockdown of HK2 suppresses OSCC cells growth, invasion, and glucose metabolism

**Figure 4:**
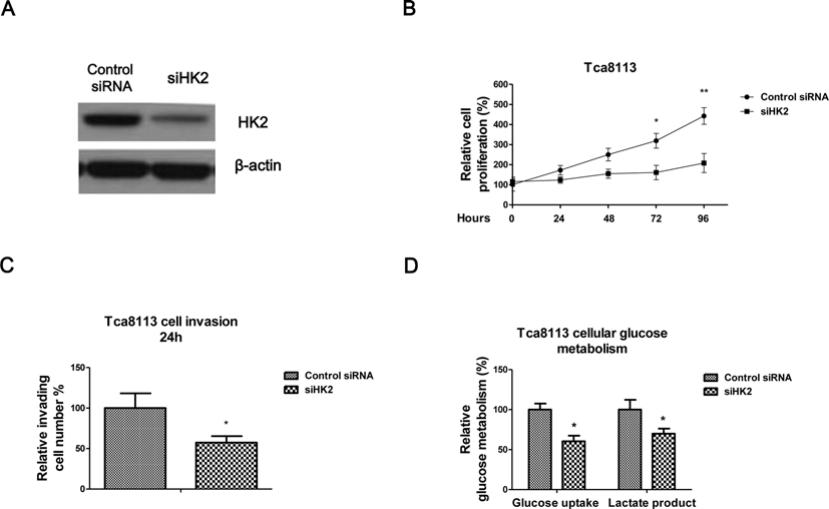
Knockdown of HK2 inhibits proliferation, invasion, and glucose metabolisms of oral cancer cells (**A**) Tca8113 cells were transfected with control siRNA or siHK2 for 48 h, cells were collected and subjected to Western blot analysis. β-Actin was a loading control. (**B**) Tca8113 cells were transfected with control siRNA or siHK2 for 48 h, then cell proliferation assay was performed at 0, 24, 48, 72, and 96 h. (**C**) The transwell assay was performed for the measurements of cell invasion ability in control or *miR-143* mimic transfected Tca8113 cells. (**D**) The glucose uptake and lactate production were measured in control or *miR-143* mimic transfected Tca8113 cells. Data are presented as mean ± S.D.: *, *P*<0.05; **, *P*<0.01.

To investigate whether the *miR-143* mediated OSCC cell suppression through HK2, we specifically knocked down HK2 by siRNA (Figure 4A). Consistently, knockdown of HK2 in Tca8113 cells significantly suppressed cells proliferation (Figure 4B), invasion (Figure 4C) and glucose metabolism (Figure 4D) compared with control siRNA transfection, indicating deregulation of HK2 by *miR-143* might be a therapeutic approach for OSCC tumor treatments.

### Restoration of HK2 rescues the *miR-143*-mediated inhibitory effects on OSCC cells

**Figure 5:**
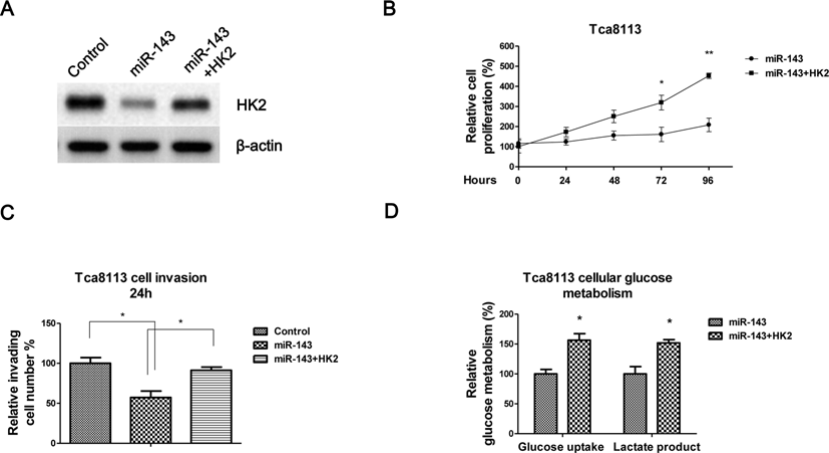
Restoration of HK2 rescues oral cancer cells proliferation, invasion, and glucose metabolism (**A**) Tca8113 cells were transfected with control, *miR-143* mimic or *miR-143* mimic plus HK2 for 48 h followed by Western blot analysis. β-Actin was a loading control. (**B**) Tca8113 cells were transfected with *miR-143* mimic or *miR-143* mimic plus HK2 for 48 h, then cell proliferation assay was performed at 0, 24, 48, 72, and 96 h. (**C**) The transwell assay was performed for the measurements of cell invasion ability in control, *miR-143* mimic or *miR-143* mimic plus HK2 transfected Tca8113 cells. (**D**) The glucose uptake and lactate production were measured in *miR-143* mimic or *miR-143* mimic plus HK2 transfected Tca8113 cells. Data are presented as mean ± S.D.: *, *P*<0.05; **, *P*<0.01.

To validate whether the tumor-suppressive function of *miR-143* was through direct inhibition of HK2 in OSCC cells, an HK2 ectopic expression vector was transfected into Tca8113 cells which were pre-transfected with pre-*miR-143*. The restoration of HK2 in *miR-143* overexpressing cells was measured by Western blot (Figure 5A). As we expected, rescue of HK2 in *miR-143* overexpressing cells led to marked enhancement of the cellular proliferation (Figure 5B), invasion (Figure 5C) and tumor glucose metabolism (Figure 5D). Taken together, the above data further supported that *miR-143* inhibited OSCC cellular proliferation, invasion and glucose metabolism through direct targeting HK2.

### *miR-143* suppresses glucose metabolism and HK2 expression in a xenograft mouse tumor model

**Figure 6:**
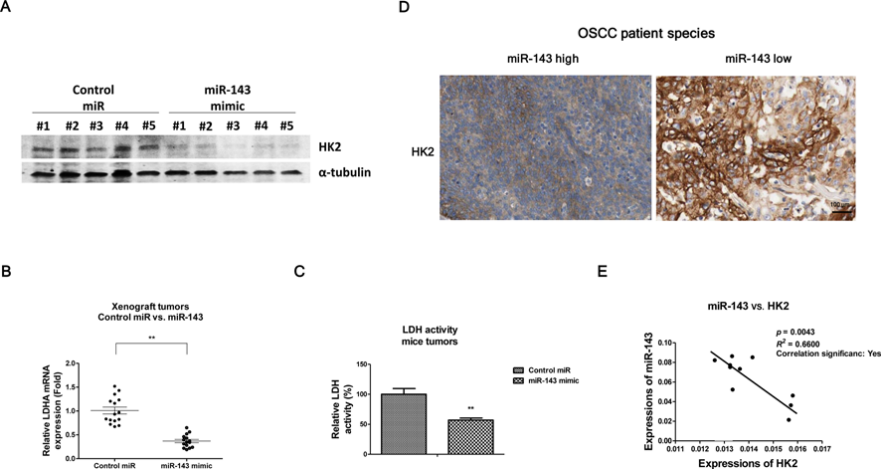
Invert correlation of *miR-143* and HK2 in OSCC patient species The mice tumors from xenograft experiments were collected and subjected to (**A**) Western blot analysis, (**B**) measurements of the mRNA expressions of LDHA and (**C**) LDH activity. (**D**) The representative expressions of HK2 in *miR-143* high and low OSCC patient species. (**E**) The correlation of HK2 mRNAs and *miR-143* levels in OSCC tissues was shown. *P*<0.05 is considered statistical significance.

To further support the above *in vitro* results that *miR-143* suppresses oral cancer cells glucose metabolism through targeting HK2, we performed *in vivo* xenograft experiments. OECM-1 cells were inoculated into the mammary fat pads of nude mice to develop tumor. After tumors formed, *miR-143* mimic or control miRNA was injected into the tumors twice a week for 2 weeks. As shown in Figure 6A, injection of *miR-143* mimic significantly down-regulated HK2 expression in nude mice. Moreover, the mRNA levels of LDHA (Figure 6B) and activity of LDH (Figure 6C) were consistently decreased in the *miR-143*-injected mice tumors. Taken together, these *in vivo* results further support that *miR-143* is a tumor suppressor through suppression of glucose metabolism.

### Invert correlation between *miR-143* and HK2 expressions in human OSCC tissues

In light of our observation that overexpression of *miR-143* led to the down-regulation of HK2 in OSCC cells, we postulated an inverse correlation between *miR-143* expression and HK2 in metastasis oral tumor tissues. As we expected, we detected a strong negative correlation between *miR-143* and HK2 in oral tumor tissues: the expression of HK2, which is associated with tumor aggressiveness [17] was up-regulated in high metastasis oral tumor species (Figure 6D) compared with low metastasis tumors, indicating that HK2 may be associated with metastasis in OSCC; in contrast, the expressions of *miR-143* were significantly down-regulated in high metastasis tumors (Figure 6E), indicating *miR-143* might be a therapeutic target for the treatments of metastasis OSCC tumor by targeting HK2.

## Discussion

Abnormal expressions of miRNAs have been implicated in the pathological processes of a variety of human cancers [6–8]. Overexpression of oncogenic miRNAs or down-regulation of tumor suppressor miRNAs plays an essential role in tumorigenesis. *miR-143* has been reported as an under-expressed miRNA in various tumors [9–14,17–20]. In the current study, we identified a tumor suppressive role of *miR-143* in human OSCC. We found *miR-143* was significantly down-regulated in oral cancer patient specimens and oral cancer cell lines. In addition, overexpression of *miR-143* suppressed oral cancer cell proliferation, migration, and invasion. Our results are consistent with the previously described functions of *miR-143* in human cancers.

HK2 converts glucose to glucose-6-phosphate, which is the first committed step in anaerobic glycolysis [21]. It has been widely studied that HK2 regulates tumorigenesis and migration in multiple cancer types, yet the mechanisms are still poorly defined [22]. HK2 has been reported to be a transcriptional target of HIF-1, which is induced in response to hypoxic conditions [23]. We identified HK2 as a direct target of *miR-143* in oral cancer cells and tumor patients, suggesting inhibition of HK2 by *miR-143* might be a new therapeutic approach for the treatments of oral cancer.

Cancer cells predominantly generate ATP as well as metabolic intermediates by a high rate of glycolysis in the cytosol, rather than by a relatively high efficient approach through oxidation of pyruvate in mitochondria [24]. In the present study, we reported the glycolysis rate was suppressed by *miR-143*, supporting the tumor suppressive roles of *miR-143* in oral cancer cells. In addition, we demonstrated restoration of HK2 in *miR-143* overexpressing oral cancer cells could increase the tumorigenesis and invasion of oral cancer cells *in vitro*. However, the mechanisms for the HK2-mediated tumor migration and invasion are still under investigation and more functional targets of *miR-143* in OSCC require further discovery. In summary, we report a tumor suppressive role of *miR-143* in human oral cancer cells and oral tumor patients. Overexpression of *miR-143* inhibited cancer cells proliferation, migration, invasion, and glucose metabolism through direct targeting HK2. Restored HK2 expression in *miR-143* overexpressing cells exhibited oncogenic effects *in vitro*. Our data suggest an important role of the *miR-143* mediated glycolysis in the development of gene therapy for OSCC.

## Abbreviations

OSCC: oral squamous cell carcinoma
HK2: hexokinase 2
LDH: Lactate dehydrogenase
HIF-1α: Hypoxia inducible factor-1α
